# Epidemiological and therapeutic studies of camel mange in Fafan zone, Eastern Ethiopia

**DOI:** 10.1186/s13071-015-1228-0

**Published:** 2015-12-01

**Authors:** Teka Feyera, Petros Admasu, Ziad Abdilahi, Bahar Mummed

**Affiliations:** Department of Parasitology and Pathology, Jigjiga University, College of Veterinary Medicine, P. O. Box 1020, Jigjiga, Ethiopia; Department of Biomedical Sciences, Jigjiga University, College of Veterinary Medicine, P. O. Box 1020, Jigjiga, Ethiopia; Regional Animal Health Investigation and Diagnostic Centre, Jigjiga, Ethiopia; Department of Microbiology and Public Health, Jigjiga University, College of Veterinary Medicine, P. O. Box 1020, Jigjiga, Ethiopia

**Keywords:** Camel, Epidemiological, Fafan zone, Therapeutic, Ivermectin, Diazinon, *Sarcoptes scabiei*

## Abstract

**Background:**

Camel mange is an economically important parasitic disease affecting productivity in camel rearing areas of the world if appropriate treatment is not instituted.

**Methods:**

A cross-sectional and a controlled field trial were carried out to study the epidemiology of camel mange in Fafan zone, Eastern Ethiopia, and evaluate the efficacy of ivermectin and diazinon in the control of mange infestation in camels on the basis of clinical and parasitological evidence, respectively. Three groups of naturally infested camels and one group of healthy camels each composed of 6 individuals were enrolled: the two infested groups received either ivermectin or diazinon, and the other groups remained untreated.

**Results:**

The overall prevalence rate of mange in camels in the study area was 31.5 % and the only identified species was *Sarcoptes scabiei*. The prevalence rate was found to significantly vary (*p* < 0.05) in relation to body condition and herd size of camels. Both drugs showed significant variation (*p* < 0.05) on improving clinical and body condition scores. Clearance of mange lesions occurred with both drugs; however, re-infestation was observed in diazinon treated group. Ivermectin significantly improved (*p* < 0.05) both body condition and clinical scores whereas diazinon markedly improved only the later.

**Conclusion:**

In conclusion, camels in the study area harbored considerable level of *S. scabiei* which warrants institution of an integrated control approach by administration of ivermectin while also sanitating the animal environment.

## Background

Camel (*Camelus dromedarius*) is an important livestock species in the pastoral economy [1], and is commonly distributed in subtropical dry areas in Africa and Asia [2]. Ethiopia is one of the largest camel populated countries in the world. With 1,102,119 numbers of camels, Ethiopia ranks third in Africa next to Somalia and Sudan [3]. In arid and semi-arid areas which are not suitable for crop and animal production, camels are superior to all other livestock in terms of food security serving as the main source of milk, meat and draft power [4, 5]. Camel is also a financial reserve and plays an important role in social prestige and wealth. However, despite its significant contribution to the livelihood of the pastoralist society, the camel is one of the neglected domestic livestock by the scientific community [6].

Slow reproduction cycle, high calf mortality and health problems are major constraints in increasing camel herd population and productivity. Ectoparasites (mites, ticks and insects) of the camel and their capacity to disease transmission are important constraints to productivity and performance [7]. Camel mange, an extremely contagious ectoparasitism caused by the parasitic mite *Sarcoptes scabiei* and transmitted by direct or indirect contact, is one of the most important parasitic diseases affecting camel [8].

Significant economic loss of camel productivity to mange has been recorded [9, 10]. Despite these facts, there is very little systematic research on mange and other ectoparasites of camels in Ethiopia. Few researchers have made attempts of reporting common ectoparasites affecting camel in different districts of Eastern Ethiopia [1, 2]. However, information on ectoparasitic infestation of camels, especially mange, in Fafan zone is inadequate despite the comparatively large number of these animals in the area, and little attention has been devoted so far to control of mange in local camels. The present work was, therefore, carried out to improve knowledge of the epidemiology of mange in camels and to evaluate the efficacy of ivermectin and diazinon for control of camel mange under field conditions.

## Methods

### Description of the study area

The study was carried out in selected districts of Fafan zone, Somali Regional state of Ethiopia. The zone is situated at 620 km southeast of Addis Ababa, the capital of the country. It has eight districts, namely Jigjiga, Kebribeyah, Harshin, Babile, Awbare, Gursum, Tullu Guled and Gololchen. Temperature of the area is generally high all the year round with mean minimum and maximum values being around 20 and 35 °C, respectively. The mean annual rainfall is 660 mm and bimodal. The camel population of the zone is 81,221 [11]. The livelihood of the community is based mainly on pastoralism (34.1 %), agro-pastoralism (56.8 %) and sedentary production systems (9.1 %) [12].

### Study design and period

The study was carried out from November 2014 to April 2015. A cross-sectional study design was employed for the epidemiological study and an experimental study design (controlled field trial) was used for the therapeutic study on selected camel herds.

### Epidemiological study

#### Study population and sample size determination

Babile, Kebribeyah and Gursum districts were selected based on high camel population, accessibility and convenience. Six kebelles/peasant associations (PAs), two from each district, were identified. Then, camel herds were selected using simple random sampling method [13], whereas subjects within herd were selected using systematic random sampling method [14]. Animals above 1 year old were included in this study irrespective of their sex, body condition score and husbandry condition. Camel herds were visited and sampled early in the morning before being released to field, and samples were then taken to Jigjiga University Veterinary Laboratory Centre.

The sample size was determined based on the formula recommended by Thrusfield [15] as follows:

$$ \mathrm{N}=\frac{(1.96)^2*\ \mathrm{Pexp}\ *\ \mathrm{q}}{{\mathrm{d}}^2} $$

Where,

N: sample size required
Pexp: expected prevalence
q: 1-Pexp
d: desired absolute precision

Sample size determination was based on 25.9 % expected prevalence according to Abebe [2] with 5 % precision and 95 % level of significance. Accordingly, the sample size required for this study was 295. The proportional probability to size approach [16] was used to determine the number of camels to be included from each district. Analyzed risk factors included: sex, age, body condition score, origin and herd size.

#### Examination for mange mites

Skin scrapings from clinically suspected cases of mange were collected and preserved in 10 % formalin. 10 % potassium hydroxide (KOH) was then added to the sediment to digest or clean the scraped material of skin, hair and other debris, so that mites were released from scabs. Finally, the specimens were carefully placed on slides for microscopic examination (40× or 100× magnification). Identification of the mange mite species was based on the morphological characteristics described by Urquhart et al. [17].

#### Age determination

Age was determined by dental eruption according to Khan et al. [18]. Camels were divided into two age categories (>3 year and ≤ 3 year) based on their puberty profile [19].

#### Body condition scoring

The body conditions of sampled camels were categorized as good, medium and poor according to Faye et al. [20]. Assessment was made on days 0, 7, 14, 21, 28, 42 and 56 for all groups.

#### Therapeutic study

This controlled field trial was carried out on camels naturally infested with mange mites. After conducting the epidemiological survey, four camel herds were selected for the therapeutic study based on their appropriateness for the experiment. This was based on communities’ complaints on intensity of the disease, personal observations during the field work, accessibility, number of affected camels in the specific village, and owners’ compliance. Overall, three groups of naturally infested camels and one group of healthy (uninfested) camels each composed of 6 animals were enrolled. Grouping was by random selection of the animals. During the therapeutic trial, camels in all groups were kept under traditional management system with similar husbandry facilities extended to them. They were allowed for free grazing in feed abundant areas usually mixed with other livestock.

#### Clinical examination and scoring of skin lesions

All camels were subjected to whole body examination for clinical signs of mange (erythema, pruritus, alopecia, hyperpigmentation and crusting) and clinically scored according to a system previously applied to horses with chorioptic mange [21]. Briefly, blinded clinical dermatological assessments (severity of the lesions and degree of recovery if any) were taken on days 0, 7, 14, 21, 28, 42 and 56 for all groups and scores were recorded as follows: 0: no clinical signs; 1: mild signs and high degree of recovery; 2: moderate signs and degree of recovery; and 3: severe signs and low degree of recovery.

#### Parasitological examination

Prior to enrollment in the study, the camels were tested for presence of mites (larvae, nymphs and adults) in skin scrapings obtained from at least 3–4 sites. Scrapings (see above) were performed on days 0, 7, 14, 21, 28, 42 and 56 of treatment at those sites where suspected lesions were present, and in selected healthy sites (the head, base of the neck, mammary gland, prepuce and flank) most likely to yield mites.

#### Therapy protocols

The camels with typical lesions of mange, harboring mites, were divided and randomly assigned by coin toss into 3 groups, and for comparisons, a fourth group of healthy camels were also included. Two miticidal agents, ivermectin (Ivomec, Change Qiankuma Veterinary Pharmaceutical, Co. Ltd., China) and diazinon (Diazinol, E.C, Company Kat Relzayat, Pesticides and Chemical, Co. Ltd., Egypt) were chosen for this field trial based on their commercial availability, patronage by camel herders and veterinary clinics in the study area, and recommendation from literature. Camels in group I received diazinon 10 days apart at a concentration of 0.1 % (spray), whereas camels under group II received two doses of ivermectin 10 days apart at dose rate of 0.2 mg kg^−1^ of body weight (subcutaneous injection). Group III (infested) and group IV (healthy) were left as positive and negative control with no treatment applied. The efficacy of each regimen was evaluated on the basis of clinical and parasitological evidence on day zero (day of treatment) and on days 7, 14, 21, 28, 42 and 56 post-treatment.

### Ethical approval

Ethical approval was obtained from Directorate of Research, Publication and Technology Transfer Research Ethics Committee, Jigjiga University, Ethiopia.

### Statistical analysis

For both the epidemiological and therapeutic studies, data was organized, edited and analyzed using statistical package for social sciences (SPSS), Version 20. The prevalence of mange was assessed using descriptive statistics. To assess differences in the prevalence and frequency of mange and association of potential risk factors (sex, age, body condition, herd size and origin) with the prevalence, the Chi-square (*X*^2^) test was used. For the therapeutic study, results and data generated from the trial were expressed as mean ± standard error. One way analysis of variance (ANOVA) and the student *t*-test was employed for inter-group and intra-group difference analysis. Results were deemed statistically significant if *p* ≤ 0.05 at 95 % confidence intervals.

## Results

### Epidemiological study

The present study revealed that camels of the study area are remarkably exposed to mange caused by sarcoptic mites*.* An overall prevalence of 31.5 % was recorded (Table 1). *S. scabiei* was identified as the only mite species in all skin scrapings collected from the suspected lesions of examined camels. Analysis results revealed no statistically significant difference related to origin, sex and age categories of the studied camels. As opposite, herd size and poor body condition score were significantly (*P* < 0.001) and positively associated to mange prevalence.

**Table 1:** Prevalence of mange infestation in camels of the study area, on the basis of different risk factors

Variable	Category level	Number examined	Number positive (%)	X^2^	*p*-value
	El-bahay	45	17 (38 %)		
	Kollej	50	12 (24 %)		
Place of origin	Arro-aska	47	17 (36.17 %)	4.29	0.58
	Gol-marodi	46	11 (24.1 %)		
	Guyow	50	18 (32.70 %)		
	Garbo-hare	57	18 (36 %)		
Sex	Male	84	27 (32.1 %)	0.021	0.885
	Female	211	66 (31.1 %)		
Age	>3 year	223	72 (32.2)	0.245	0.620
	≤3 year	72	21 (29.16)		
	Good	110	18 (16.3)		
Body condition	Medium	148	53 (35.8)	26.34	<0.001
	Poor	37	22 (59.5)		
	<20	107	24 (22.4)	16.22	<0.001
Herd size	20–40	136	41 (30.1)		
	>40	52	28 (53.8)		

### Therapeutic study

Treatment with ivermectin and diazinon was similarly effective on sarcoptic mange infestation, and beneficial on clinical and body condition scores. Both treatments resulted in the clearance of skin of mangy camels (Table 2). No parasitic stages were found throughout the observation period post application of ivermectin. However, reinfestation was observed in a camel during the 8th week post diazinon application. Furthermore, one of the healthy untreated animals was found infested with mange at the end of the observation period.

**Table 2:** Effect of ivermectin and diazinon treatment on mange infestation in camels

Group	D0		D7		D14		D21		D28		D42		D56	
	NPA	ENM	NPA	ENM	NPA	ENM	NPA	ENM	NPA	ENM	NPA	ENM	NPA	ENM
Ivermectin	6	++	0	–	0	–	0	–	0	–	0	–	0	–
Diazinon	6	++	0	–	0	–	0	–	0	–	0	–	1	+
Infested untreated	6	++	6	+	6	++	6	+	0	+	6	++	6	++
Healthy untreated	0	–	0	–	0	–	0	–	0	–	0	–	1	+

+: 1–10 mites; ++: 10–100 mites; −: No mites; n (number of animals in each group) = 6; D = day; D0 = the day treatment commenced; NPA = Number of Positive animals; ENM = Estimated number of mites

Throughout the observation period, treatment with either drug did not result in significant improvement in the body condition score compared to pre-treatment values and amongst themselves (Table 3). Relative to positive controls, treatment with ivermectin seemed to improve the body condition score (28 through 56 days post-treatment; *p* < 0.05), whereas the diazinon group had a similar body condition score except in week 7. The positive and negative controls showed deteriorating and stable body condition scores, respectively.

**Table 3:** Effect of ivermectin and diazinon treatment on body condition score change of experimental camels

Goup	D0	D7	D14	D21	D28	D42	D56
Ivermectin	1.83 ± 0.17	1.67 ± 0.21	1.67 ± 0.21	2.17 ± 0.17	2.33 ± 0.21^a^	2.50 ± 0.22^a^	2.50 ± 0.22^a^
Diazinon	2.00 ± 0.26	2.00 ± 0.26	2.17 ± 0.17	2.00 ± 0.26	2.67 ± 0.21	2.33 ± 0.21^a^	2.00 ± 0.26
Infested untreated	2.00 ± 0.00	1.83 ± 0.17	1.83 ± 0.17	1.50 ± 0.22	1.50 ± 0.22	1.33 ± 0.21	1.50 ± 0.22
Healthy untreated	2.33 ± 0.21	2.67 ± 0.17	2.67 ± 0.17	2.67 ± 0.17	2.33 ± 0.21^a^	2.67 ± 0.17^a^	2.67 ± 0.17

Values are mean ± SEM; n (number of animals in each group) = 6; D = day; D0 = the day treatment commenced; SEM = standard error of mean; all superscripts indicate significance at *p* < 0.05 (^a^compared to infested untreated)

Treatment with either drug resulted in a considerable improvement in the clinical score compared with pre-treatment values and the positive controls (Table 4). Differences were statistically significant from the third week post treatment to the end of the trial. All treated camels showed high degree of recovery with reference to skin texture, healing of skin lesions and disappearance of crusts. However, the infested untreated camels showed worsening skin appearance compared to the treated groups.

**Table 4:** Effect of ivermectin and diazinon treatment on clinical score change of experimental camels

Group	D0	D7	D14	D21	D28	D42	D56
Ivermectin	1.83 ± 0.75^b^	1.67 ± 0.52^b^	1.33 ± 0.52^b^	1.00 ± 0.63^ab^	0.67 ± 0.52^a^	0.67 ± 0.82^a^	0.67 ± 0.52^a^
Diazinon	1.67 ± 0.82^b^	2.00 ± 0.90^b^	1.33 ± 0.52^b^	0.83 ± 0.41^ab^	0.33 ± 0.52^a^	0.67 ± 0.82^a^	0.50 ± 0.84^a^
Infested untreated	1.67 ± 0.82	1.13 ± 0.52	1.67 ± 0.82	2.17 ± 0.41	2.00 ± 0.63	2.17 ± 0.75	2. 33 ± 0.53
Healthy untreated	0.00 ± 0.00^a^	0.00 ± 0.00^a^	0.00 ± 0.00^a^	0.00 ± 0.00 ^a^	0.00 ± 0.00^a^	0.00 ± 0.00^a^	0.33 ± 0.52^a^

Values are mean ± SEM; n (number of animals in each group) = 6; D = day; D0 = the day treatment commenced; SEM = standard error of mean; all superscripts indicate significance at *p* < 0.05 (^a^ compared to infested untreated; ^b^ compared to healthy untreated)

## Discussion

The present study demonstrated an overall prevalence of 31.5 % mange mite infestation in the studied camel herds. This value is higher than the figure in Awol et al. [22], Dinka et al. [1], Lawal et al. [23] and Chaudhry et al. [24] who reported 16.7, 10.7, 3.5 and 3.14 % prevalence from Northern Ethiopia, Eastern Ethiopia, Sokoto-Nigeria and Cholistan-Pakistan, respectively and lower than a prevalence of 83 % reported by Al-ani et al. [25] in Jordan. Variation in genetics, environment, accessibility to veterinary services, herd size and other husbandry practices could justify these differences.

*S. scabiei* was identified as the only mite species in all scrapings collected from suspected skin lesions. This observation is in general agreement with reports by various authors [1, 22, 23]. Even though both sarcoptic and chorioptic mange mites have been reported, sarcoptic mange caused by *S. scabiei* is by far the most common, contagious and serious condition in camels [26, 27]. Similarly as Megersa et al. [28] and Dinka et al. [1] from southeastern and eastern Ethiopia, respectively, the prevalence of mange did not differ according to the origin, sex, and age of the camels. Not surprisingly, mite prevalence was highest in camels with poor body condition score (59.4 %). Moreover, the prevalence of *S. scabiei* increased significantly in herds with a larger size. Probably, camels living in larger herds are more prone to come into contact with infested animals, e.g., during herding, housing and suckling.

Amongst factors that may influence the condition of camels, control of ectoparasites with effective chemicals such as endectocides and ectoparasiticides is very important. These drugs can be used for the benefit of local epidemiological awareness [29]. In the present work, their efficacy was evaluated on the basis of clinical improvement and the parasitological findings. Data revealed that the clearance of skin of infested camels occurred following either treatments (with ivermectin and diazinon). Successful use of ivermectin in mangy camels at a dose rate of 200 μg/kg body weight has been reported [30]. In the present study, mangy animals treated with ivermectin felt rapidly more comfortable and docile due to quicker relief from itching.

While no lesions were found during microscopic examination of the skin scrapings up to the 7^th^ week post treatment with diazinon, re-infestation was observed at the 8^th^ week with resurgence of the typical mangy skin lesions. As opposite, the animals treated with ivermectin neither showed any lesions nor signs of re-infestation throughout the observation period after treatment. The result of the present work also agrees with the reports of Bala and Rath [31] and Abdally [29], who stated that ivermectin have good efficacy against sarcoptic mites. Sprays such as diazinon have also been used to clean the animal environment to eliminate the risk of recurrent infestation from fomites [32, 33]. Saber and Ahmed [34] suggested that using acaricides for treatment in addition to spraying animal environment (bedding, wall and fomites) is the best protocol for controlling mange in water buffaloes.

In this field trial, the treated camels were not isolated from the herd, hence they had opportunity of contact with other sick animals and a contaminated environment. It was, thus, impossible to tell how and when they became re-infested. However, the results of this study demonstrated that re-infestation may occur if treated animals are not isolated from untreated similars or infected premises and environment [35].

## Conclusion

The present study conducted in Fafan zone, eastern Ethiopia showed that camels living in this area are frequently infested with mange mites with a likely impact on their health and productivity. The overall prevalence of mite infestation was 31.5 %, *S. scabiei* being the only mite found in affected camels. Origin, sex and age were not significantly associated with the prevalence of mange mite infestation, while a positive association existed with poor body condition and herd size. The therapeutic field trial showed obvious clinical improvement in all the treated camels. The study also revealed that ivermectin was relatively more efficacious than diazinon, as measured by analyses of skin scrapings, and body condition and clinical score changes.
